# On the Physiology of Absorption and of Respiration

**Published:** 1853-07

**Authors:** M. C. Bernard


### SELECTED ARTICLES.

### ARTICLE IX.

*On the Physiology of Absorption and of Respiration.* By M. C. Bernard. Novel views, based on Experiments with vivisections respecting the rôle which oxygen and carbonic acid gases play with regard to the blood, and their relation to the production of sugar in the system. Notes* of Lectures, at present in course of delivery at the College of France, May, 1853.

In order to show the independent action of the muscular and nervous tissues, M. Bernard repeated the experiments upon frogs which he had exhibited in the private course of lectures

* As we have had to translate our notes without as careful a revision as we should have desired, in order to communicate them in time for the July issue of the Charleston Medical Journal, we must beg some indulgence for the phraseology of portions. We have, however, endeavored to render them as accurate as was in our power, by a comparison with the published authorities, when any could be found.

on physiology, delivered at the Ecole Pratique during the winter. It was demonstrated thus: Upon touching with a galvanic fork the exposed cerebro spinal nerves of one of these animals, prepared by stripping off its skin, in the course of which it was killed, there was produced the well-known convulsive contractile movements. This also took place when the muscles alone were touched. A second frog, killed by introducing under the skin a poison, which possesses the property of acting solely upon and destroying the sensibility of the nerves, gave no indications whatever of movement, when those which were exposed were touched, whilst, at the same time, the contractions of the muscles of the legs continued quite as active when the instrument was applied to their surface.

This sensibility, under the conditions expressed above, would continue to manifest itself for sometime in both animals, though it persisted longer in the second. There are other substances which when introduced under the skin produce precisely an opposite effect, viz. destroying the sensibility of the muscles, and leaving intact that of the nerves.

Of all the functions of the economy of organized life, whether vegetable or animal, that of absorption is the most universal and important. It prevails over, and gives a direction to all others. It is also extremely variable as to its activity and persistence, both with respect to individuals, to special organs, and to time. It enjoys periods of rest, and of activity, of disease and increase, dependent upon the peculiar conditions with which it is associated. In certain organs, as in the stomach of the higher animals, there are intervals when no absorption occurs, whilst at the same time the process is constant in other parts of their economy, as in the glands, capillaries, &c. In the embryo we find circulation, digestion, elimination, all dependent upon absorption, existing at one and the same epoch.

There are three principle species of absorption. 1. Ærial, as in respiration, where we have gases taken up by the lungs. 2. Absorption of liquids, of water or its vapor. This occurs in all the tissues. To live, the individual requires a certain amount of humidity. The tissues are never absolutely dry but always moistened to a greater or less degree. 3. Alimentary absorption. This is sometimes intermittent; in the higher animals, for example, there are digestive periods during which it is more active. Alimentary absorption is direct or indirect. M. Bernard avoids the consideration of other species or varieties of absorption, as that of caloric, of light, and of electricity, concerning which less is known.

Before entering into the examination of the general conditions upon which absorption depends, there are two important questions to determine. Firstly, Are all soluble substances forcibly absorbed? It has been usually supposed that for a substance to be absorbed it must first be dissolved, or be capable of dissolution, which was considered the essential pre-requisite. Secondly, Are there not certain insoluble substances absorbed? To the first question Bernard's experiments have enabled him to reply in the negative. He has ascertained conclusively that there are certain matters perfectly soluble, which, when placed in contact with animal tissues, are not absorbed, and manifest no action whatever on the economy. He was led to discover this by experimenting upon the poison of serpents. If this was introduced into the skin of an animal, so as to reach the capillaries, death would inevitably occur. The introduction of the same substance into a circulatory vessel, or swallowed, so as to come in contact with the lining membrane of the stomach, was followed by no injurious results. In the latter situation it would not operate as a poison. Some physiologists have attributed this to the antagonistic action of the gastric juice, which they say digests or renders the poison inactive by neutralizing it. Bernard addressed himself to the task of examining into the truth, and he elicited the following facts: A dog with a permanent fistulous opening, communicating with the stomach, was placed on the table. Through this was introduced a large quantity of the poison. After a considerable interval a small portion of the gastric juice, containing the poison in dissolution, was extracted, with which a sparrow was inoculated by introducing it through a small prick made into its skin. The bird died in a few minutes, showing that the poison, though mixed with the fluids of the stomach, was not absorbed, and had lost none of its active properties. To prove that gastric juice alone would not produce death, another sparrow was inoculated with a portion obtained from the dog's stomach, previously to the introduction of the poison. It was not in the least inconvenienced by it. The gastric juice, then, does not destroy or modify the poison—it is only the stomach which shows an election with respect to the absorption of certain poisons. The venomous serpent consumes and digests incessantly, as did the dog, his own poison. In eating the prey which it has killed, it carries in its stomach a liquid which contains a toxic principle of the greatest virulence, but from which the elective affinities of certain of its membranes preserves it. Bernard calls this *elective* absorption.

In pursuing this subject it became an interesting inquiry to discover in what portion of the walls of the stomach, resided this power which prevented it from absorbing certain animal poisons. By experiments with a small apparatus introduced into a vessel holding the gastric juice, which contained the poison, but from which the tube was separated by a portion of the stomach of an animal, the water alone was observed to rise in the tube, whilst the poison remained behind. But, upon removing the epithelial covering, the absorption of the poison, as well as of the water, took place, and the liquid which had penetrated the septa, formed of the portion of the stomach, was as poisonous as that below. Thus showing that it is owing to a special action residing in the epithetal coat that there is non-absorption of certain poisons.

There are substances perfectly soluble which are not indiscriminately absorbed by all tissues. M. Bernard's discoveries have shown that the veins will absorb cane sugar—that the lymphatics never absorb sugar, the chyliferous vessels will receive fat, and reject sugar, none of which is found in the vena cava, or in the extension of these vessels. Prussiate of potash is taken up by the veins and lymphatics, thus indicating a special affinity or election in the phenomena of absorption, as regards both tissues and vessels. These subjects, however, have been frequently referred to and published on other occasions, and we only allude to them as they now form interesting points in the physiology of absorption.

With respect to the second question: are substances not soluble never absorbed? The word insoluble is objectionable as it is vague, and is capable of two interpretations; a substance insoluble in water, may be soluble in another liquid. A second insoluble out of the stomach may be so when introduced by meeting there certain agents, which act upon, and thus alter, its chemical affinities. For example—an oxyd of copper, insoluble out of the stomach is dissolved by the animal matters which it finds within. So also sugar has the property of rendering silica insoluble. Chalk, silica, &c., regarded as insoluble, are thus dissolved by agents with which they come in contact in the economy. This principle, connected with the absorption of insoluble substances, is worthy of attentive consideration—for, by the presence or absence of a certain proportion of sugar, for example, we have or have not deposits, whether normal or abnormal, of the earthy matters of the economy. By the assistance of the product which they there encounter, they are dissolved, and are thus conveyed through the channels of the circulation to fulfil their part in the formation of structure of a particular kind, or, they are eliminated by the emunctories, and thus escape from the system. Experiments to show this may be performed externally, as by adding to a solution containing sugar and silica, a substance possessing greater affinity for the former when the deposit of crystals of the latter make its appearance. Sugar renders lime soluble by forming a sugar of lime.

But can any substance really *insoluble* enter into and be absorbed, by the economy, like soluble matters? It has been asserted that particles of carbon have been taken up by the lacteals and lymphatics, and have entered into the circulation. M. Bernard stated that experiments had demonstrated that this was really true. He had examined, at Clemarts, the bodies of persons who had been tattooed, and he had found the mineral substance placed under the skin, whether Prussian blue or vermilion, taken up by the lymphatic ganglia. It was always in one of the lymphatics that the particles entered, and it was necessary to prick the skin and insert it. Can this, though, be considered a true absorption? Has it not rather the character simply of a penetration or deposit? By experiments made by M. Bernard and C. Robin, at which M. Bernard also assisted, they found that when lamp black was introduced into the stomach of rabbits and birds, it was not discovered in the circulation after the most careful scrutiny with the microscope. If common powdered charcoal was substituted it was easily found in the lymphatics and in the vena cava. The latter had salient pointed angles, and he attributes the penetration of these, and not of the former, into the intestines, to this mechanical difference in their structure. So that even here it may have been rather a penetration than a bona fide absorption.

The next question to be determined is, what parts or tissues of the body absorb, and what are the substances most usually taken up? The variety respecting it is almost endless, all parts of the economy being indued with the power of absorption, whether it be, first, of gases; secondly, of fluids; or, thirdly, of solids.

As we have just observed, the phenomena of absorption are sometimes elective. A membraneous septa introduced between two gases, or liquid substances of different densities, permits of the transit of the one and the other, with different degrees of facility, varying with the substances employed—the membrane refusing a passage to one, whilst it is readily granted to another. The mucous membrane of the lungs of animals does not absorb, equally well, all æriform substances that come in contact with it, as for example, hydrogen—nor does the skin absorb, indiscriminately.

To demonstrate this, M. Bernard exhibited a rabbit, under the skin of which, at the preceding meeting, three days previously, he had forced a quantity of air, by means of an india rubber bag with a sharp mouth-piece attached. With this the skin was blown up until the animal became almost one-third more than its original size. The orifice in the skin was then closed; and upon analyzing the air—which was received in small glass tubes under mercury, in one of which was introduced a bit of phosphorus—it was found that the oxygen had been absorbed in the interval, leaving behind the nitrogen (and carbon.) The failure of the phosphorus to change the level in the tube, indicated that but one element of the air had been absorbed; and thus furnishing a conclusive proof of the elective affinities of this tissue, also, for certain gases.

In the phenomena of exhalation, also another property of certain tissues, especially of epithelial structure, the glandular organs exercise an election. The entire mucous surface of the intestinal canal, including that of the mouth, is hostile to the absorption of animal poisons, as was proved by the experiments related above. There is an exception, with respect to a portion of the mucous membrane of the lung, which, strange to say, will readily absorb the same poison, and, of course, with a fatal result to the animal.

As this fact is exceedingly interesting, in a medical point of view, and in its relation to the constitution of the atmosphere, M. Bernard, before proceeding to demonstrate it by experiment, entered into the consideration of the influence of oxygen upon the air, in respiration, and upon the blood.

The absorption of oxygen is indispensable to life, and hence, by some, it is called vital air, par excellence. Vegetables also, equally with animals, require it, though in them the process is inverted, as they exhale it during the day and absorb it from the under surface of their leaves during absence of light. It is consumed by them, however, in great quantity. In the germination also, of plants, the absorption of oxygen is indispensable. The embryo of the vegetables (as well as that of the animal) absorbs this gas, and will refuse others. If you place the eye or young shoot in a small tube or receptacle, containing oxygen gas, its absorption is evident; but if this gas be replaced by any other, the absorption is arrested.

To be absorbed, oxygen also retires within the great law that it is soluble, and can therefore be capable of dissolution. If you introduce it into the vein of an animal, it is asserted that it can be seen in the shape of small bulbæ or vesicles, and Spallanzani stated that he discovered them in the circulation of frogs. But air never enters in this way, in the circulation of man. If you introduce it in great quantity in the venous system of an animal, it produces death by obstruction, for the bulbs or vesicles of air pass with difficulty, if at all, through the cells of the lungs. If introduced forcibly and quickly into the jugular vein, as was done by Magendie, it mixes with the blood and goes directly to the aorta; but it cannot pass through the lungs unless incorporated with the blood—therefore, when thrown in in too large a quantity, so that its dissolution could not take place, the animal died. The obstruction is mechanical, and the result indicates that to be inocuous, its complete absorption into the blood is necessary. Oxygen has been frequently introduced into the arteries, by forcing it through a small opening made by the mouth of a syringe, and after a certain time it was found to have been absorbed, for had it not been dissolved and been taken up by the blood, it would have passed on to the capillaries, and have produced gangrene.

But oxygen gas is very readily absorbed by blood. As experiments prove, it far surpasses water in its power of absorbing oxygen. 1000 volumes of blood, agitated in the air, absorb 130 of oxygen; 1000 volumes of water absorb but 9¼*. Now if you add atmospheric pressure, you modify its absorption by water: but this has very little influence upon the blood†. Liebig said that the globules alone absorbed, but there is no doubt that the serum does also, though to a less degree. Some animals have no red globules, hence these are not exclusively endowed with this power, however, it may be readily acknowledged that, in vertebrates, their principal rôle is the absorption of oxygen.

But is the oxygen absorbed by the blood chemically combined with it, or is it a state of dissolution purely simple?

When we add certain substances, as phosphate of soda or carbonic acid to water, they increase its capacity for absorption.

*Observations of Gay Lussac, see Magnus' Annals of Physic and Chemistry, 1846—v. lxvi, p. 177. See also Chimie Anatomique et Physiologique, by Robin and Verdeil, published in 1853, vol. ii, p. 36.

†Nouvelle's Lettres sur la Chimie, trad. France, 1852—Paris, p. 89.

This is true also of a salt of iron. Liebig affirms that the saline matter of the blood likewise has a special affinity for oxygen, and retains it in solution. But there are objections to these explanations which are entirely chemical—one of which is the great ease with which the oxygen of the blood will be given up for other gases, as nitrogen for example—or if placed in relation with carbonic acid, when there is an immediate interchange.

M. Bernard acknowledges, however, that these are not conclusive. There is another proof still more interesting in favor of the idea of its chemical combination. If it was a dissolution purely simple, it should augment under pressure—if, for example, one exposes carbonic acid gas to water under pressure, the relative amount absorbed is very much increased. In blood, on the other hand, a very immaterial influence is produced upon the volume of oxygen absorbed under pressure. To the quantity expressed above, in the 1000 volumes of blood, the same quantity of water there is an increase by 4 in each, which is by no means in the same proportion. There is an evident *disproportion* between the two substances in this respect. An individual on a high mountain under pressure of the atmosphere, will absorb more than ordinarily; but here, as in exercise also, the influence of oxygen absorbed does not change materially, or even sensibly his feelings. The oxygen, by the very affinity of the blood, for it can enter easily into, and is readily taken up by, that fluid, so that persons can sustain, without inconvenience or material difference of feeling, varying degrees of atmospheric pressure, and respire air, more or less charged with oxygen, as Regnault and Reiset's experiments proved.*

The bearing of the above, on what follows, will be seen; but there is another question in relation to certain changes which oxygen undergoes and in reference to its reception into the circilatory system of man and animals, which must be considered.

It is well known that oxygen is believed to undergo certain changes in a nascent state, by which it is endowed with powers

***Sur la respiration des animaux, (comptes rendus des Soc. de L'Acad. des Sc. de Paris, 1848, p. 25.)**

not belonging to it ordinarily. Freme and Becherel attributed it to its contact with electricity, and Schonbein, who discovered ozone, as it is called, also observed the influence of electricity in the atmosphere, in inducing its presence. Under these conditions, decomposition would take place of the iodides of potash with starch, which would indicate its presence.

By adding also certain substances to air, it was found to be altered in character, and ozone was the result. This ozone is very irritating, and can scarcely be breathed—when animals are forced to respire it, bronchitis and pneumonia are induced. From this and other reasons, Schonbein was inclined to attribute influenza and grippe to its presence in the atmosphere. He has since written a second memoir, in which he persists in the opinions first expressed, and treats more fully of the rôle which it fills in the modifications of atmospheric air in certain seasons and localities, during the existence of epidemics. He says that it modifies the presence or quantity of malaria, and is inclined to unite the well known fact of the purification of the air by electricity to the power it possesses of producing ozone. M. Schonbein also conducted a series of experiments on the influence which electricity has in hastening the decomposition of flesh. Ozone on the other hand retards the putrefaction of viands, which, when removed from contact with it, decomposed more rapidly, and hence he considers it a purifier of the atmosphere.*

So that oxygen does acquire certain modifications under certain conditions, by which it becomes endowed with new powers. Cannot oxygen also, when received into the lungs be absorbed and digested, as we may say. Alimentary matter during digestion acquires new powers and undergoes important changes.

*M. Bernard did not refer to it, but we may cite also a very interesting paper by Mr. Faraday, who made experiments in London on the electrolysis of water, and on the product called ozone, in which he described it as an intermediate state of oxygen. It was republished in the annual report on the physical sciences for the year 1852, and we believe in the London, Edinburgh, and Dublin Journals, of the Medical and Physical Sciences. The reader is also referred to papers on the connection between the existence of ozone and the presence of cholera, in the Western Lancet.

The question, therefore, is a natural one, whether oxygen also does not submit to alterations, and obtain new attributes, when placed in contact with the tissue of the lung or the blood itself? Is it identically the same afterwards as it was in the air?

Dumas said that absorbed oxygen suddenly underwent modifications, by which it acted differently within than without—but this has not been conclusively settled, and the object of M. Bernard's researches will be to determine some of these very questions, respecting the introduction of this gas into the system—to know whether it is changed or simply absorbed with the establishment of no new relations between it and the living body.

In order to determine this, let us study the mechanism of absorption as it exists in different organs and in different parts of the system, by this means ascertaining the different modes of its reception.

*Influence of Oxygen upon the Phenomena of Life.*—Lavoisier stated that a man would absorb 1075 grammes in twenty-four hours. Now, what is the mechanism of this absorption? There is always a membrane between the blood and the oxygen, viz., the interposed membrane of the lung. How does the oxygen of air, which is external, pass within? You will find it in the blood itself when examined. Has the blood, therefore, a chemical or physical influence upon the oxygen? Is it only simply to the solubility of oxygen, or, as some believe, to a special affinity? There is certainly a species of attachment on the part of the blood for the oxygen, and its absorption continues incessantly.

M. Bernard will endeavor to trace out the circumstances which favor the absorption and introduction of it into the blood. Recent experiments of his, upon the substance of blood, which he repeated before us, have contributed to throw considerable light upon the subject, and at the same time furnish analogies which may reflect it upon others.

As the capacity of blood for the absorption of oxygen was greater than that of water, it was attributed to something which it contained. It has been also observed that sugar added to the facility of absorption of liquids in which it was dissolved, and, therefore, it was supposed that its presence in the blood might account for its greater power in this respect. These experiments, to demonstrate this, were performed out of the body, and were neither sufficient nor conclusive. Bernard, as he will proceed to show, has ascertained that exactly the reverse is true. In physiology it is essential that they be confirmed by experiments on living animals. Bernard, therefore, presented us with blood which he had drawn, on yesterday, directly from the jugular vein of an animal, with a small syringe, which did not permit it to come in contact with the external air, and he had passed it under quicksilver into two graduated tubes of glass, containing each the same quantity of oxygen gas. In one of these was introduced a small portion of sugar of the grape. The tubes were divided into parts of 1-10 of a millimetre, and the amount of blood introduced was exactly the same in both, being at 83°.

In tube *M*, not containing sugar, in which the blood was introduced on yesterday, 14 divisions of oxygen were absorbed immediately: 21 additional divisions, absorbed to-day, upon agitating the tube the sugar being now completely dissolved. Total 35.

In tube *N*, containing sugar, 11 divisions were absorbed on yesterday, and 8 divisions were absorbed to-day. Total 19. So that the one containing sugar has absorbed but 19 parts, whilst the other, with no sugar, has absorbed 35 parts. Observe what a material difference.

M. Bernard examined in the same way, but without introducing sugar, the blood freely drawn from different portions of the body, and in comparing the results he found that they varied in a manner very distinctly in proportion to the amount of sugar which exists in the blood, in different portions of the body.

If you wish blood containing very little sugar, take it from the vena porta. That holding most sugar is found in the subhepatic veins. Upon examining these, the blood of the former absorbed a much larger quantity than that from the latter, all of which, of course, confirm the original experiment.

Lavoisier taught that in the process of pulmonary respiration, the carbon combined with the oxygen and formed carbonic acid. This is now universally accepted. But the carbonic acid is found under certain circumstances and in certain individuals, not derived from the inspired air. It is already formed in the blood, and the oxygen in entering, simply displaces it. A frog, for example, which was forced to breathe hydrogen gas, exhaled carbonic acid, so that it came from his body, and at any rate gave indications that the ordinary respiration in the lungs was not the exclusive source of its formation.

Some have also asserted that the amount of carbonic acid exhaled or expired, always loses a certain and definite relation to the amount of that and oxygen gas inspired. Now it has been proved by modern observers, among others by Regnault and Reiset, that the proportion varies—the quantity of oxygen absorbed being greater during abstinence and less during digestion relatively also to the amount of carbon exhaled. These observers, in establishing the diminution in the relative power of certain organs to absorb oxygen at different periods, were not acquainted with the development which M. Bernard is now prepared to give to their observations, and hence the testimony of each contributes to strengthen the other. Bernard is now competent to answer the question, why is it that the amount of oxygen absorbed is more considerable during abstinence? It is owing to the fact that the blood then contains less sugar. Digestion is an intermittent function, during which the amount of sugar present in the blood varies considerably, being, however, always increased, upon which, as we have seen above, the capacity or non-capacity of the blood to absorb, very much depends.

Now, in abstinence, the vena porta acts very much like other veins; it then contains little else than the blood from the intestines brought by the mesenteric veins.* The hepatic veins and the vena cava have also very little at this period. During di-

* For the extended explanation of this, see previous reports of M. B's researches on digestion, and on the abdomino renal circulation. We had drawn these out to accompany this paper, from notes taken at his course of lectures in December, but as they have been published by others, will retain them.

gestion on the contrary, the vena porta has a double rôle to perform, in addition to the venous blood it carries to the liver, the alimentary matter eliminated from the small intestines. And it is of this that the liver fabricates the sugar which is found at this period in large quantity in the sub-hepatic veins. So that there are moments when the blood holds the largest amount of sugar, there being then what may be called its summum of intensity. And therefore, from what has been stated above, there must necessarily be oscillations in the proportion of oxygen absorbed, depending as it has been shown, upon the presence or absence of sugar.

These facts, having the collateral testimony of Regnault's observations, which were carefully made, support M. Bernard's idea respecting the influence of sugar, which, by the external demonstration on freshly drawn blood, we have seen to modify considerably the absorption of oxygen.

Even in the incubative state, as was before remarked, there is absorption—all living animals, the vegetable embryo, the egg as well as the fetus absorb oxygen. The fetus of some, as of mammifers, absorb it even when not in contact with the external air. In the fetus of mammals, including man, the action of the placenta is analogous to that of the lung—but the process is not like that produced by the contact of air with the lungs, and blood of the mother.

For in the fetus, the blood differs from that of the mother; there is no distinction between that in the arteries and veins, and before respiration occurs, the color is the same in both umbilical vein and arteries. Bischoff and Valentin said that it was intermediate, but their observations were obtained from animals which had made some inspirations which were quite sufficient to induce alterations in its appearance. In the fetus, as Fourcroi proved, the venous blood when mixed with oxygen, did not turn red as did that of adults, but it gradually acquired this property as greater advances to maturity were made. In reality, the blood of the mother differs essentially from that of the fetus. But as soon as it acquires the power of exercising its proper functions, that is, those of ordinary extra-uterine existence, by which oxygen is absorbed immediately; the liver also commences to perform its peculiar functions, and the condition of things change. The fetus has no need for oxygen; the liver is well developed, and it fabricates sugar in considerable quantity—in fact, all fetuses are diabetic.

Bernard tested this frequently at the Abattoirs of Paris, on a number of animals. The sugar was found in great quantity in the urine. As soon, though, as pulmonary respiration commences, the relations are altered, oxygen is absorbed, and the blood becomes florid. So that digestion is only *magazining* by certain organs for the requirements of the blood primarily, which ultimately ministers to the general demands of the system.

At certain periods, during digestion in animals with long extended intestines, the urine becomes very turbid—whereas, in an opposite state it is clear. Now there are diseases, induced by derangements of nutrition, as in diabetes, where the urine is charged with sugar, and which, as M. Bernard supposes, may be attributed to the diminished capacity in the blood for the absorption of oxygen.* As we are aware, he is now able to render animals artificially diabetic, by a process which acts directly upon the functions which regulate the production of sugar by the system.

In cholera, also, Rayer has remarked that the quantity of oxygen absorbed was very much less.

It has been an object with some, to determine what relations there are between the amount of oxygen absorbed, and that eliminated with the carbonic acid, and it was supposed that what was exhaled was in proportion to that absorbed. At present, we should be convinced that this is not correct.

The following figures, derived from other observers, particuly from Regnault and Reiset,† M. Bernard presented for con-

* Mr. Wurtz referred a few mornings since, in his lectures on organic chemistry, to the plan pursued at the Hotel Dieu and elsewhere, of giving patients affected with this disease, bread which contained nothing but gluten, which will thus furnish less sugar to the system than that made in the usual way. A specimen was exhibited, which was very much lighter than the ordinary bread.

† See work cited above.

sideration, as they contribute materially to strengthen his views, founded upon experiments made by himself.

In a rabbit fed by Regnault on carrots, which contain sugar, during full digestion, there was, of parts of air respired, 100; of carbonic acid-gas exhaled, 91.9; amount of oxygen absorbed and which remained in the blood, 8.1.

In the same animal during abstinence, of 100 parts, there were 69.0 exhaled as carbonic acid, and 31.0 of oxygen absorbed. This variation depends upon the increase or decrease in the amount of sugar in the blood, which has been shown to be so constant during these two different states. In a dog fed on viands of 100 parts, 75.2 of carbonic acid gas was exhaled, and 24.3 of oxygen absorbed. In the same animal fed upon bread, the figures were 100, 91.3, 8.1. In the last example the food resembles more closely that given to the rabbit, and we can observe the relations. The third essay was to notice what effect would ensue from the introduction into the system of any substance which would completely destroy the secretion of sugar, in order to ascertain if the results would be constant. Bernard had ascertained that the digestion of fat had this property; for no sugar can be made by means of this introduced into the system. This experiment, on this point, exactly confirmed the others, which were derived from other observers. Upon feeding a dog in good health on fat, the numbers stood thus: 100—69.4—30.6; evidently showing its alliance with the phenomena noticed in the cases cited above.

Are there any other substances, which, if introduced into the blood would produce a similar effect, or an opposite? There is one found normally in blood, in nature, and in almost all the tissues of the body, chloride of sodium—common salt—which acts in a manner exactly opposite to that of sugar. It augments the power of the blood to absorb oxygen. Bernard tried the same comparative experiments with this, in contact with blood placed in graduated tubes, and they were followed by the general results, exactly the reverse of those which ensued upon the introduction of sugar, in similar tubes, in contact with freshly drawn blood, kept from the influence of external air. In the tube which contained the salt and blood, the following proportions presented themselves: 100—68—32. In the second tube, containing blood and pure oxygen were derived, 100—80—20, the middle figures representing as before, the carbonic acid exhaled, and those on the right, the oxygen. So that the two phenomena are in a measure inverse—the quantity of oxygen gas absorbed being increased, and the amount given off as carbonic acid diminished when chloride of sodium is employed.

No one had made experiments upon chloride of sodium to ascertain the modifications it induces upon respiration and the blood. M. Bernard has thus ascertained, as the above experiment shows, that in animals its reception into the system leads to changes in the amount of oxygen absorbed by the lungs, precisely opposed to those which result when sugar is substituted. When salt enters in great quantity into the food of animals, there is a considerable increase in the absorption of oxygen, over those which do not feed on it. Bernard made experiments on man and animals, and Reder (?) and Malgaigne on horses. If a very slight quantity is given, then the effect is not marked; if it be increased, the appetite augments, a great quantity of urea is found in the urine, together with chloride of sodium, there being much more of the latter also present than ordinarily.

Bernard had ascertained that during digestion, the urine of animals contained a comparatively small quantity of urea, and that the alkali and carbonates were augmented. After an abstinence of twenty to thirty-eight hours, these disappeared, and the secretion again became acid—so we observe that when there exists the largest amount of oxygen in the blood, there is more urea in the urine—and thus the phenomena are allied together, and as the oxygen augments in adding salt to the food received into the system, it is easy to compare them. Common salt then has an influence upon the absorption of oxygen by the blood, in a ratio inverse to that of sugar.

Bossingault had examined the subject under another point of view. He found that an animal having salt mixed with its food, differed from one not receiving any into the system, in fattening more rapidly. It stimulates the appetite, and consequently assimilation and nutrition, for the increased amount of oxygen absorbed contributes to the requirements of its system. Respiration is more active and so is digestion. But if the quantity is increased very largely, it grows thinner than the other, because when thus added to excess, the animal is forced to consume a portion of its own blood, as is the case with carnivorous animals when fed exclusively on herbs. Chloride of sodium, when administered to an animal, not only increases the amount of oxygen absorbed, but the rapidity with which it is affected becomes much exalted. The 32 volumes for example in the experiments referred to above, were absorbed immediately.

Bernard has not yet been able to come to any satisfactory results, as to the effect produced by these agents upon the globules, themselves, of the blood. He has only determined that under the influence of chloride of sodium, these become smaller and flatter than a natural process of exosmosis taking place. In extending the examinations to obtain the effect produced by sugar, no material differences were observed.

With respect to the influence of sugar upon the nutrition of animals, his experiments were affirmative. Sugar, with feculent substances, possesses very marked powers in fattening. It gives rise to the formation of sugar in the blood, and consequently prevents too much combustion by oxygen. Fat alone, will not do the same, as Magendie proved very conclusively, by feeding animals on it exclusively. They lost in flesh, and grew lean. To render fat beneficial to the economy, certain conditions are essential, and one is its combination with sugar which assist by the influence the latter possesses, upon the absorption of oxygen. Chloride of sodium acts adversely by destroying too much oxygen, which, as we are now aware, passes out in combination with carbonic acid in much larger quantity than when sugar is employed.

Medicines, M. Bernard adds, had sometimes an effect upon the respiration of individuals, and we will be able after a while to trace their influence more in detail, and with more precision by observing in what way they are related to the facts just observed. Its explanation may possibly, also, be more readily ascertained. Does absorption and exhalation occur in other surfaces or by other organs? Frogs are known to absorb oxygen by the skin, and there is probably exhalation of carbonic acid, by this surface also—their lungs in addition performing their ordinary functions. Is it not a constant fact, that any membrane which can absorb one will exhale the other gas. The skin of mammifers including man, does exhale, we are aware, a certain amount of carbonic acid, (but does not absorb oxygen.)* Experiments on horses, show also that the urine often contains an increased quantity of carbonates. This was illustrated by testing the urine of a rabbit placed on the table, which gave abundant effervescence when hydrochloric acid was added to it. At another period it presented an entirely different re-action. But more of this anon. Some observers have said that an acid exists in the lungs, which acted on the oxygen of air and produces the carbonic acid. Carbonic acid being much more soluble than oxygen, it is thus more easily held in solution by the blood which furnishes it. This constitutes a second mode of explanation.

Tiedeman and Gmelin accounted for the phenomena by the supposition that the oxygen gave rise to acetic acid in the lungs, and this acting upon the carbon contained in the blood, freed that portion which passed out as carbonic acid.

Upon carefully injecting cyanuret of mercury into the femoral artery, it was found in the returning veins not at all decomposed or changed, giving out its usual characteristic odor. If on the contrary it was forced into the pulmonary artery the animal was poisoned, because the lungs decomposed it—a chemical action took place and prussic acid was formed. So that there is a distinction between the action of the capillaries and that of the lungs. M. Bernard thinks it is occasioned by the acid in the latter tissue, being analogous to the changes produced by sponge platinum on certain substances. It is not a vital

* Spallanzane, demonstrated that it was a property of all organized matter! living or dead, to absorb and fix oxygen as well as to disengage carbonic acid. See Robin and Verdeil, Chimie Anatomique et Physiologique, page 39, vol. 1, Paris, 1853.

property of special tissues, for a bit of lung, produces externally, when placed in contact with it, the same decomposition of the cyanuret of mercury in giving rise to hydrocyanic acid.*

Verdeil has just discovered a new acid in the lung which crystallizes, and which he calls pneumic acid, to which he attributes the power of decomposing carbon or of displacing the carbonates of the blood. So that some are authorized in saying, that the acid which is subservient to the elimination of the carbon, is dispensed by the lung. It is secreted, M. Bernard says, in the cellules of the lungs.

Another theory held on this subject is entirely physical, which is, that the carbonic acid gas existing in the blood, is retained there by a certain pressure which is removed upon reaching the lungs, and upon which it escapes. But the blood does not hold it, they say, as a chemical combination, but in solution just as water can be made to contain a greater amount of this gas by subjecting it to pressure; when it is removed, as for example, when one raises the cork of a bottle of eau de seltz, the carbonic acid instantly escapes. It is supposed that the right ventricle may have the power of forcing it into the blood.

But this is simply an opposition in the condition of things, and not an explanation. Blood drawn from an individual, in which case the pressure is removed, does not lose the acid.

Bernard is of the opinion, that these theories are unnecessary. You must seek in the properties of the blood itself, for its power of retaining carbon and oxygen. The blood can contain various gases, but it never disengages them spontaneously. It never disengages oxygen spontaneously even under a pneumatic apparatus. They must always be extracted by displacement in submitting it to some other gas. Thus, if you place it in a pneumatic apparatus in contact with hydrogen, in giving place to it, it will lose the gas which it previously held. The same general rule is applicable to liquids—these can only lose

* Author with Ch. Robin, of the Traite de Chimie Anatomique et Physiologique, Normale et Pathologique, in 3 volumes, with plates, just published. Refers to Bernard's views and the influence the acid may have on the decarbonization of the blood.

one of their component elements by receiving a different one. Exosmose and endosmose, must concur. Dufresnoy established this, and it is equally true of both gases and liquids.

When one injects oxygen gas under the skin of a rabbit, as Bernard did before us, the same phenomena occur. After a while the oxygen was found to have changed its properties. It was precisely like the process of exhalation going on in the lungs, the quantity of the gas was not materially diminished, but when it was collected in a tube, and examined, it was found to have been converted into carbonic acid. Before a bit of potash was placed in the graduated tube in which this was received, it occupied a position indicated by the figures, 98 D, it fell down to 88 D, after its introduction.

Atmospheric air introduced under the skin of the rabbit and allowed to remain there for twenty-four hours, was represented by these figures: 5.4° carbonic acid, 3.5.

Pure hydrogen introduced under the skin of the rabbit, when withdrawn and examined after twenty-four hours, was represented thus: 4.0 (Cos.)

The air in the lung comes in contact with blood containing the three gases, oxygen, carbonic acid, and a small quantity of nitrogen.

The membrane lining of this organ, has the special property of allowing these gases to pass, and the blood that of permitting one to escape when another enters without itself transuding.

Now, under the skin of the anamal, the same conditions exist under a somewhat different form; by means of the capillary vessels, there is a disparation or absorption of oxygen, and the apparition of carbonic acid. Both in the lung and under the skin, it is the oxygen which displaces the carbonic acid. The more we examine the economy at large, the more we will bring the general fact to hold good, that when there is an entrance of one, there is the corresponding exit of another.

But it is not in the membrane of the lung that the blood finds the gas. The blood containing the one, finds itself in contact with that contained in the external air, just as if no membrane separated them. Besides, there is a certain amount of porosity pertaining to the structure of the lung. But this specialty of the pulmonary membrane, strange to say, does not exist in all parts of the organ, which is extremely interesting in a medical point of view. Magendie thought, that the mucous membrane of the bronchia and the cells, was not continuous or identical in structure, but that they terminated abruptly. At present, it is found to extend throughout, but the differences are distinctly marked. The pulmonary parenchyma or the cells have not the same structure with the membrane which lined the bronchial tubes. Certain poisonous substances, miasma, gases, and air, are only absorbed by the former and not by the latter. The microscope now shows us that it is the epithelium, lining these two portions respectively, which differs as materially in structure as they are found to vary in their physiological properties. Bernard had exhibited experiments, a few days previously, to show us that the mucous membrane of certain portions of the air passages, as well as that of the stomach with which it was continuous, would not absorb certain poisons *(curare)* which were quite soluble. The mucous membrane of the bladder, of the eye, and other organs, were also hostile to their absorption, but it was otherwise with that lining the extremity of the air passages. These poisons proved noxious when allowed to come in contact with the membrane composing the ultimate ramifications of the bronchial tubes, and the air cells. These distinctions are applicable to the contagious matter of the plague, and to noxious volatile airs, &c., which on the other hand can be readily received into the skin.

To demonstrate the above, a rabbit was placed on the table, and suffered no inconvenience from a bit of *curaré* introduced into the stomach. An opening was then made into the trachea and a small portion was placed in contact with the internal lining of the bronchia by means of a tube extending some distance down. As soon as a sufficient time was allowed (about three to ten minutes) for the poison to be dissolved and to reach the pulmonary vesicles, death occurred. It is observed to be attended with none of the usual cries and evidences of suffering which that caused by poisons, administered in other ways, always gives rise to. It is absolutely necessary for the poison to reach the lung cells before the slightest evidence of inconvenience is manifested, and death then occurs easily, but with certainty. The trachea and bronchia alone, exhibited no power whatever to take up the poison, although it was dissolved by the mucus from their surfaces in order to descend into, and reach the membrane lining the cells.

To illustrate the influence of the inhalation of oxygen gas by the lung, a rabbit was placed upon the table, in an inverted jar which communicated by one pipe, with an apparatus which supplied it with oxygen gas, and by another with a basin containing quicksilver, which permitted the experimenter to ascertain the composition of the air which escaped.

Under these circumstances there will be a manifest alteration in the composition of the urine.

In digestion the urine of an animal is found to be alkaline, containing a large quantity of the carbonates in dissolution—upon adding sulphuric acid, there is effervescence with the disengagement of carbonic acid. The same animal with this acid secretion, if kept fasting, will lose the carbonates of the urine which will become alkaline, and be found to contain uric acid in such considerable quantity, that in some animals, as in the horse, it crystallizes.

Now, these alterations, so constant and so diametrically opposed to each other, depend entirely upon the different amounts of *oxygen absorbed* by the blood during digestion, and when the animal is not in digestion.

The examination of the changes, which occurred in the urine of the rabbit before and after the experiment, were directly confirmatory of this as well as the principles before stated, respecting the entire dependence of these inverse phenomena upon the composition of the blood, and the character and amount of the ingesta to be absorbed.

*It is the blood which respires, and not the lungs, exclusively.* Let us examine then, into this property of blood, which is related so closely to the sugar, salt, &c., which is produced in the system. We are, therefore, interested in knowing whether the blood in all parts of the body has the same capacities, and in the same degree. This fluid obtained from different parts of the economy, is not found to be the same, for after passing through, and depositing in each, proportions varying in character with the varying demands, and the peculiarities of structure and function it necessarily loses certain of its elements. If we examine consequently the blood which has supplied muscles, a gland, the brain, &c., &c., we find it presenting well marked differences.

There must, under every circumstance, be contact of blood with oxygen. In the lung, the mucus membrane is very thin and delicate, and the microscope indicates an essential difference between the epithelium of the cells, and that of the bronchia. Those of the former, are simple and polygonal in form, whilst the latter are vibratile, much thicker, more elongated, and endowed with motion, the cilia having an upward direction, by which the mucus, secreted by the glands and particles of foreign bodies, tend externally to the orifice of the respiratory passages.

This specialty of the ciliary membrane, can be seen in the mouth and stomach of dogs and frogs. By opening the mouth, esophagus and stomach, longitudinally, small particles of carbon placed at one extremity, are seen to be carried throughout its whole extent, until they reach the other. These ciliary movements persist even in the cadaver for forty-eight hours, but strange to say they are instantly arrested by anæsthetics.

Now, independently of this respiration by the lungs, no one has said that other parts of the body can and do perform the same functions. Bernard not only asserts that it is necessary that the blood should respire in other and different parts of the body, but that the blood of certain organs must respire differently as well as absorb differently, varying with the peculiar condition of each. The physical as well as physiological distinctions existing between them, are well marked. In respiration you must have extended contact of blood with air, but this is not all, for the blood itself is not, throughout, equally apt to absorb.

If you define respiration "the absorption of oxygen and the escape of carbonic acid," then the skin respires, for, as before remarked, in a general way, it has been demonstrated that this occurs in that organ.

If one plunge the arm in a vessel containing oxygen gas, it will be observed to diminish, and you can analyze and compare it with that obtained from the lung; in the course of five hours 1-5 will be found to have disappeared, as oxygen and carbonic acid has been exhaled in its place.

If you plunge the arm under cold water, also, bulbs are soon formed, which rise to the surface, and which it has been asserted are composed of carbonic acid.

M. Bernard said that there was certainly the alteration and disparation of a certain quantity of the oxygen. In the subcutaneous cellular tissue as the experiment with the rabbit under whose skin air was introduced proved, the air had experienced modification very similar to what occurs to that which passes through the lungs. If you inject air, as Nysten (?) did, into the peritoneum or the pleura, the oxygen disappears, and carbonic acid occupies its place. The figures obtained by this observer differ from those of others, but still the fact is incontestable that the same phenomena are visible.

In other animals, other tissues besides the lungs respire as in birds, where there is respiration through the air cells in the cerous membranes of the bones. There might be chemical causes for this greater respiration in these animals, but in the lungs and pleura there is the same absorption of carbon and oxygen—the proportion varies, but the phenomena are constant.

The respiration of muscles has not been heretofore determined.

It is important to determine whether these phenomena are analogous to pulmonary respiration. Many physiologists said that they were not, being nothing more than "an absorption of one, and the replacement of the other." Bernard differs from them.

M. Guerlat (?) has performed a number of experiments upon man, dogs and horses, to prove that the skin respires. He employs a species of air tight vessel, like a cupping glass with a stop-cock attached, which permits the enclosed air, which has been acted upon by the skin over which it has been placed, to pass out, and to be analyzed. He always found that there had been a disparation of a certain quantity of oxygen and the apparition of carbonic acid, but he also observed that the proposition of the former was very slight in reference to the amount of exhalation of the latter, as these figures show 100 vols.—1.99 carbonic acid exhaled, 0.86 oxygen absorbed.

These experiments were applied to different portions of the body, and in the different animals, and the conclusions just referred to, were obtained, so the facts are true. But is it a veritable respiration?

Guerlat said that in arresting this action of the skin, as you can do by covering it with varnish, the animal, a rabbit for instance, died. Magendie accomplished the same by covering them with a solution of gelatine or oil. The temperature always diminished and the animal was said to die of cold. Guerlat said by asphyxia.

Bernard thinks, there is something in addition to pure asphyxia, in which the venous blood cannot become arterial. He thinks, in fact, that phenomena take place exactly inverse to those characterizing the respiration in the lung. In other words, that the blood of the arteries and veins in varnished animals, instead of being black as in asphyxia, ordinarily is on the contrary, red in both vessels. The arterial blood in an animal in this condition in passing through the capillaries does not become black, and hence the changes are inverse to what occur in pulmonary respiration.

This peculiarity depends upon a special cause. There is a special office of the capillaries in all the organs, by which the arterial blood is changed to venous, and this is under the influence of the nervous system. If, as Magendie did, you sever clear across the entire thigh of an animal, leaving only the two large arterial and venous vessels uninjured, after a quarter of an hour you perceive that the blood of both artery and vein is red. The destruction of the nerves have paralyzed the action of the capillaries and their functions are so deranged as not to be able to be manifested. Now, if these nerves be galvanized, the capillaries resume their work, and the blood becomes black as ordinarily. Again, if you administer to animals certain powerful poisons, as prussic acid, which acts directly and exclusively upon the nervous system, the blood of the veins, and that of the left side of the heart, does not blacken. So that this respiration is not purely physical; there is something else than simple contact of blood and oxygen—the action of the capillaries is as important and as marked as that of the special power residing in the lung cells, and they depend upon the integrity of other portions of the system. Besides the blood, in different parts and organs of the body, acts differently. These members for example were obtained by three experiments upon that of the carotid artery, and two upon that of the vena porta—they represent the relative amount of oxygen absorbed. Carotid artery, 13.10, 12.8. Vena porta, 21.9, 23.2. So that respiration is affected even in the termination of the arteries, though it is of course not so active from obvious causes.

In the frog, even the blood which has traversed the skin is arterial, whilst in man it is venous, because in the former, that which reaches the skin, passes directly from the heart—the skin indeed possessing a membrane penetrated by vessels very analogous in structure to that of the lung.

[We must conclude for the present, but we may say that M. Bernard proceeds to show that the respirating process in the blood, as well as the animal heat of different parts of the body, depend upon the influence, and consequently the integrity of the cerebro spinal system.]—*Charleston Med. Journ. & Review.*

